# Ovalbumin and Kappa-Carrageenan Mixture Suppresses the Oxidative and Structural Changes in the Myofibrillar Proteins of Grass Carp (*Ctenopharyngodon idella*) during Frozen Storage

**DOI:** 10.3390/antiox10081186

**Published:** 2021-07-26

**Authors:** Noman Walayat, Xiukang Wang, Asad Nawaz, Zhongli Zhang, Abdullah, Ibrahim Khalifa, Muhammad Hamzah Saleem, Bilal Sajid Mushtaq, Mirian Pateiro, José M. Lorenzo, Sajid Fiaz, Shafaqat Ali

**Affiliations:** 1 Department of Food Science and Engineering, College of Ocean, Zhejiang University of Technology, Hangzhou 310014, China; 2 College of Food Science and Technology, Huazhong Agricultural University, Wuhan 430070, China; zhangzhongli@webmail.hzau.edu.cn; 3 College of Life Sciences, Yan’an University, Yan’an 716000, China; 4 Jiangsu Key Laboratory of Crop Genetics and Physiology, College of Agriculture, Yangzhou University, Yangzhou 225009, China; 007298@yzu.edu.cn; 5 College of Biosystems Engineering and Food Science, Zhejiang University, Hangzhou 310027, China; 0619344@zju.edu.cn; 6 Food Technology Department, Faculty of Agriculture, Benha University, Moshtohor 13736, Egypt; Ibrahiem.khalifa@fagr.bu.edu.eg; 7 College of Plant Science and Technology, Huazhong Agricultural University, Wuhan 430070, China; saleemhamza312@webmail.hzau.edu.cn; 8 State Key Laboratory of Food Science and Technology, School of Food Science and Technology, Jiangnan University, 1800 Lihu Avenue, Wuxi 214122, China; 7180102916@stu.jiangnan.edu.cn; 9 Centro Tecnológico de la Carne de Galicia, Avd. Galicia n°4, Parque Tecnológico de Galicia, San Cibrao das Viñas, 32900 Ourense, Spain; mirianpateiro@ceteca.net (M.P.); jmlorenzo@ceteca.net (J.M.L.); 10 Área de Tecnología de los Alimentos, Facultad de Ciencias de Ourense, Universidad de Vigo, 32004 Ourense, Spain; 11 Department of Plant Breeding and Genetics, The University of Haripur, Haripur 22620, Pakistan; sfiaz@uoh.edu.pk; 12 Department of Environmental Sciences and Engineering, Government College University Allama Iqbal Road, Faisalabad 38000, Pakistan; shafaqataligill@gcuf.edu.pk; 13 Department of Biological Sciences and Technology, China Medical University, Taichung City 40402, Taiwan

**Keywords:** myofibrillar protein, cryoprotective effect, oxidative stability

## Abstract

This study was done to analyze the cryoprotective influence of ovalbumin (OVA) with kappa-carrageenan (KC) in grass carp myofibrillar proteins during frozen storage. Ca^2+^-ATPase activity of MP was significantly reduced due to protein denaturation and showed a direct association with decreased sulphydryl (SH) contents and tertiary structural properties. Besides that, an increase in carbonyl, surface hydrophobicity, and dityrosine contents was observed. The addition of OVA-KC significantly restricted the decline in Ca^2+^-ATPase and SH groups, which were further confirmed by the retarded increase in carbonyls. Furthermore, the addition of OVA-KC increased the stability of α-helix contents. Moreover, MP treated with 6% OVA-KC also improved intermolecular interaction forces linked with gelling and water holding properties of MP. Therefore, it can be concluded that OVA-KC could be used as an effective cryoprotectant in fish and related products for preservation and commercialization.

## 1. Introduction

Grass carp (*Ctenopharyngodon idella*) is one of the major freshwater fish species in China due to its low prices, better yield, and growth [1]. The improved nutritional and processing qualities make this fish more valuable for storage and commercialization [2]. It is used in fresh as well as in processed form [3] and even its by-products are also used [4,5]. However, during the post mortem stage—due to rigor mortis and intensive stress—alterations are critical for an adequate transport and storage [1,6]. Frozen storage is the most effective and widely used approach, which can preserve the nutritional profile of fish muscles by inhibiting water, enzyme, and microbial activity [7,8]. However, crystallization, oxidation, protein denaturation, and other adverse factors can reduce the quality of fish products as these remain unavoidable during frozen storage [2].

Myofibrillar proteins (MP), which include myosin, actin, troponin, and other proteins, are the main components of fish muscles, covering 65–75% of total proteins [9]. MP are susceptible to temperature fluctuations and can be quickly oxidized during frozen storage. Furthermore, key factors that influence the structural properties of MP include crystallization, lipid oxidation, and partial dehydration [10]. In addition, quality properties, including functional and processing attributes, are significantly affected by oxidative alterations that occur during freezing in proteins [11]. The above-mentioned alterations that typically occur in MP within first two months of storage [12]. During frozen storage, oxidative modifications are primarily responsible for the decrease in flavor, gelling, and textural characteristics of final product. These changes are driven by MP oxidation and alteration in intermolecular covalent interaction forces [7]. Additionally, the increased dityrosine, sulfhydryl’s reduction and Ca^2+^ activity in ATPase can also lead to oxidative changes in MP [13]. Furthermore, oxidative alterations in MP decrease protein binding and increase the coagulation of myosin molecules, leading to a coarser gel [14,15].

Cryoprotectants (sugar and sorbitol) are generally incorporated to avoid fish protein oxidation during frozen storage. The basic aim is to provide a protective shield to proteins against aggregation and dehydration by interacting with different amino groups. However, sucrose and sorbitol could produce excessive sweetness and increase caloric values, which would be inappropriate with the latest consumption trend of low sugar balanced foods [16]. 

Ovalbumin (OVA) has become one of the effective additives in aquatic products owing to its high nutritional and antibacterial effects. Moreover, OVA can also be used as an efficient emulsifier and gelling agent, which can act as an enzyme inhibitor to prevent the functional losses in surimi products [17]. In this regard, OVA has been reported as an effective antioxidative agent due to the presence of sulfhydryl groups and strong binding abilities with metal ions. Besides, after contact with saccharides, the antioxidant effects of OVA are surprisingly enhanced.

κ-Carrageenan (KC), extracted from red seaweed belong to the class *Rhodophyceae*, is a sulphated linear polysaccharide made from subunits of 3,6-anhydrogalactose. The application of KC has been confirmed in the food industry since it enhances gelling, water retention, and thickening properties [10]. Moreover, KC can also increase the structural and functional properties by inhibiting the free water molecules [11]. During frozen storage conditions, KC has been reported to be an important antioxidant in the protection of MP. KC can establish complex gelling networks by filling interstitial species between protein molecules and improving the viscoelastic properties of gel through the interactions with functional sites. Therefore, in this research, the effect of OVA-KC was analysed in the MP of grass carp during 60 days of frozen storage. This study also deals with the functional, conformational, and intermolecular interaction forces between the MP and OVA-KC, especially focusing on the potential antioxidant properties. Moreover, it sheds light on the application of OVA-KC as an effective additive and as a low sweetness cryoprotectants in the prolonged commercialization of fish and related products during frozen storage.

## 2. Materials and Methods

### 2.1. Materials

*Ctenopharyngodon idella* (length: 35 ± 2 cm, weight: 2.7 ± 0.3 Kg; *n* = 12) was purchased from the local fish market (Wuhan, China). The live fish was immediately transported to the laboratory and slaughtered. Fish mince, cleaned from bones, was used for MP extraction. OVA was purchased by Aladdin Company (Shanghai, China). KC and other chemicals were purchased from Sinopharm Chemical Reagent Co., Ltd. (Shanghai, China).

### 2.2. Modification of OVA by KC

Different concentrations of OVA and KC (0, 2, 4, and 6%) were prepared by mixing uniformly and magnetically stirring at 25 °C for 2 h at a constant low speed and water bath temperature. The OVA-KC was obtained and stored at −4 °C for further use. 

### 2.3. Extraction of Myofibrillar Proteins

MP from *Ctenopharyngodon idella* was prepared according to the method of Zhang et al. [10] ith minor modifications. Minced was rinsed and homogenized (XHF-DY, Ningbo Scientz Biotechnology Co, LTD. Ningbo, Xinzhi, China) 5 times with 0.1 mol/L Na_3_PO_4_ buffer solution at 3500 r/min followed by centrifugation (Heraeus Multifuge X1R centrifuge, Thermo Fisher Scientific, Osterode, Germany) at 10,000× *g* at 4 °C for 10 min. The obtained precipitates were homogenized and centrifuged with the same buffer solution twice. After that, the MP pellets were washed twice with five volumes of 0.1 mol/L NaCl using the above-mentioned homogenization and centrifugation conditions. Later on, connective tissues were removed from the MP using 4 layers of cheesecloth. The protein concentration was 75.4 mg/mL determined by the Biuret method using BSA as a standard curve [12].

### 2.4. Preparation of Samples

Next, the extracted MP was equally weighted and divided into four parts. The MP samples were added into the 1000 mL beakers and different concentrations of OVA-KC were incorporated and mixed with spatula. After that, all MP samples were filled into the storage tubes (50 mL), tightly capped and stored at −18 °C for 60 days. MP without additives was used as a control sample.

### 2.5. Determination of Protein Solubility

The MP was centrifuged (10,000× *g*, 10 min, 4 °C) with 10 volumes of high salt buffer solution. Protein content was determined from the obtained supernatant. The percentage of protein content was determined by the biuret method after and before centrifugation. The solubility of the protein product was calculated as

Protein solubility (%) = (Protein content after centrifugation)/(Protein content before centrifugation) × 100%(1)

### 2.6. Determination of Carbonyl Content

The carbonyl contents were determined with the method of Nikoo, et al. [18] with minor changes. MP (2 mg/mL) was diluted with TCA 10% (*w*/*v*), and centrifuged at 2000× *g* for 10 min. The sediment solution (500 μL) was added into the tubes along with 2 g/L of 2 mol/L DNPH in HCl. Samples were left in darkness for 1 h at 36 °C. The precipitates were received after centrifugation (2000× *g*, 10 min, 4 °C), washed with ethanol: ethyl acetate (1:1, *v*/*v*) and centrifuged at same conditions again. The centrifuged pellets were added with 6 mol/L of guanidine (2 mL) followed by 2 mol/L of HCl. The prepared mixture was incubated at 37 °C for 15 min and centrifuged. The absorbance was measured at 365 nm against the controls. The carbonyls formation was calculated through an absorption coefficient (22,000 (mol/L)^−1 cm^^−1^) and expressed as nmol/mg of proteins.

### 2.7. Determination of Ca^2+^-ATPase 

Ca^2+^-ATPase activity of MP with OVA-KC was determined according to the method of Liu, et al. [14] with some modifications. MP (3 mg/mL) was mixed with 0.2 mL of reaction solution. Then, 1 mL of TCA (100 g/L) was added to stop the reaction and centrifuged the mixture at 2500× *g* for 5 min and 4 °C. The obtained supernatant (1 mL) was mixed with ammonium molybdate (3 mL) in 0.75 M H_2_SO_4_ and 100 g/L of FeSO_4_ (0.5 mL) in 0.15 M H_2_SO_4_, which were properly mixed with the above prepared mixture. The change in Ca^2+^-ATPase activity was analyzed using the inorganic phosphate at 700 nm. All samples were taken and examined in triplicates and expressed as nmol of phosphate.

### 2.8. Determination of Sulfhydryl Content

The sulfhydryl (SH) content of OVA-KC MP was analyzed according to the method of Ellman [16] with some changes. MP was mixed with a high salt buffer solution and adjusted to 5 mg/mL. Then, MP (0.5 mL) was added to 4.5 mL of a buffer solution (pH = 7.0) composed of EDTA (10 mmol/L), NaCl (0.6 mol/L), Urea (8 mol/L), SDS (20 g/L) and centrifuged at 10,000× *g* for 10 min. The supernatant was mixed with 0.5 mL Elleman reagent (0.2 mol/L Tris HCl, 1% DTNB). The mixture solution was placed in a water bath at 40 °C for 25 min. The absorbance was measured with a UV-spectrophotometer (tu-18, Beijing Pukenye General Instruments Co., Ltd., Beijing, China) at 412 nm. The results were calculated using an extinction coefficient of 13,600 M^−1^ cm^−1^ as shown in the equation

SH (mol/(〖10〗^5 g) proteins) = (A_412 × D)/(13,600 × C) × 〖10〗^4(2)

where D is the dilution of MP and C is the concentration of MP (mg/mL).

### 2.9. Determination of Dityrosine Content

Dityrosine content was determined according to Nikoo et al. [13] with slight modifications. MP samples (5 mg/mL) were added with high salt buffer solution and properly mixed. The mixture solution was centrifuged (5000× *g*, 10 min, 4 °C) and the obtained supernatant was measured with a Fluorescence Spectrophotometer (F-4600, Hitiachi High Technologies Co., Japan) using the following conditions: excitation wavelength (325 nm), emission (420 nm) with slit width (10 nm). All results were taken and analyzed in triplicate. The calculated results are expressed in arbitrary units (A.U.).

### 2.10. Surface Hydrophobicity

Surface hydrophilicity of MP treated with OVA-KC was conducted according to Lv, et al. [17] using bromophenol blue (BPB) bonding method with some modifications. MP samples were adjusted to 3 mg/mL by addition of 20 mM of phosphate buffer (pH-6.0). MP samples (1 mL) were added with 200 µL of bromophenol-blue (10 mg/mL) and mixed well for 10 min with a shaker at room temperature. The mixture was centrifuged at 2000× *g* for 15 min. Then, the obtained supernatant was diluted with phosphate buffer (1:10, *v*/*v*) and the absorbance (A) was determined at 595 nm using BPB as control. All results were calculated through the following equation and expressed in bound-BPB.

BP bound (µg) = 200 × (A_(595 control) − A_(595 sample))/A_(595 control)(3)

### 2.11. Fluorescence Intensity (FI)

Tertiary structural changes of MPs were determined using Fluorescence Spectrophotometer (F-4600, Hitiachi High Tech., Co., Tokyo, Japan) following the method described by Walayat, et al. [19] with minor modifications. MP (0.05 mg/mL) was added with 0.6 M NaCl and measurements were obtained at an absorbance range of 300–450 nm, slit 10 nm, excitation and scanning speed were 295 nm and 1000 nm/min, respectively. NaCl (0.6 M) was used as control. All samples were analyzed in triplicates and mean values were reported. 

### 2.12. Circular Dichroism (CD) Spectrum

MPs secondary structural properties were analyzed with a circular dichroism spectropolarimeter (J-1500-150, JASCO Co., 192-8537, Tokyo, Japan) using the method of Lina, et al. [20] with slight modifications. MPs samples (0.05 mg/mL) were added with 0.6 M NaCl and CD spectrum was determined through quartz cuvette (10 nm), spectrum range (200–250), resolution (1 nm), response time (2 s), scanning speed (10 nm), and sensitivity (50 millidegree). All α-helix, β-turn and random values were calculated and expressed in molecular ellipticity [θ] deg cm^−2^ dmol^−1^. Meanwhile, all secondary structural values were generated by CD in percentage. All samples were analyzed in triplicates to determine the mean values. All samples were run against NaCl (0.6 M) as baseline.

### 2.13. Intermolecular Interaction Force

Intermolecular interaction forces were determined following the procedure described by Pérez-Mateos, et al. [21] with slight modifications. MP 2 g was added with 20 mL of NaCl (0.6 mol/L, pH = 7.0) solution (S1). The mixture was homogenized at 4000 r/min for 2 min and placed at 4 °C for 1 h. The homogenized mixture was centrifuged at 10,000× *g* for 25 min and 4 °C. The obtained supernatant was stored at 4 °C for 1 h. The obtained supernatant from S1 was added with 20 mL of Urea (1.5 mol/L) + NaCl (0.6 mol/L, pH = 7.0) solution (S2). The mixture was homogenized and centrifuged at the same conditions mentioned above and the supernatant was stored at 4 °C for 1 h. The collected supernatant from S2 was again homogenized and centrifuged with 20 mL of Urea (8 mol/L) + NaCl (0.6 mol/L, pH = 7.0) solution (S3) and the supernatant was stored at 4 °C. After that, the supernatant was homogenized and centrifuged with 20 mL of β-mercaptoethanol (0.5 mol/L) + NaCl (0.6 mol/L) + Urea (8 mol/L, pH = 7.0) solution (S4) using the same conditions. In the end, the protein content of each supernatant was measured by the Lowry method [22], where S1 = ionic bonds; S2 = hydrogen bonds; S3 = hydrophobic interactions, and S4 = disulfide bonds. All samples were taken in triplicates and expressed as mg/mL.

### 2.14. Statistical Analysis

Statistical analyses were performed using SPSS software (SPSS 21.0, SPSS, Chicago, IL, USA). Normal distribution and variance homogeneity had been previously tested (Shapiro–Wilk). The data were submitted to one-way analysis of variance (ANOVA). Duncan’s test was also performed to calculate the significant differences among samples (*p* < 0.05). All experimental analyses were carried out in triplicates and reported as mean value ± SD.

## 3. Results

### 3.1. Protein Solubility

Protein solubility is an important indicator to evaluate fish and fish products quality. The decline in protein solubility mainly occurs due to protein denaturation, aggregation, and unfolding of protein molecules [2]. Moreover, changes in intermolecular bonding could also be related to ionic, hydrogen and hydrophobic interactions and water-soluble proteins [23]. In addition, these factors are generally associated with decreased functional, gelling and water holding properties [24]. The decline in the protein solubility in control and OVA-KC samples is shown in Table 1. During the frozen storage, the protein solubility was significantly (*p* < 0.05) reduced in all MP samples. At the beginning of storage, there was no noteworthy difference between the samples analyzed. The significant drop (*p* < 0.05) in protein solubility started on day 15 of frozen storage. The control sample showed a prominent decline from day 0 to day 60, which was almost similar to that observed in samples treated with 2% of OVA-KC. Although, 4% OVA-KC and 6% OVA-KC also showed substantial declines, these were less pronounced than those observed in control and 2% OVA-KC samples. MP treated with 6% OVA-KC exhibited the best results after 60 days of frozen storage, with a considerable stability against oxidative changes compared to the other samples treated with OVA-KC. Zhang et al. [10] stated that the addition of carrageenan oligosaccharide inhibited the decline in protein solubility in white leg shrimp than the samples added with sodium pyrophosphate and control. The decline in protein solubility could be due to the migration of water to extracellular spaces resulting in reduced textural and water binding properties. Therefore, the results obtained from the current study showed that increasing the concentration of OVA-KC can reduce the decline in protein solubility by inhibiting protein aggregation, denaturation, and the formation of strong intermolecular binding interactions.

### 3.2. Carbonyl Content

**Figure 1:**
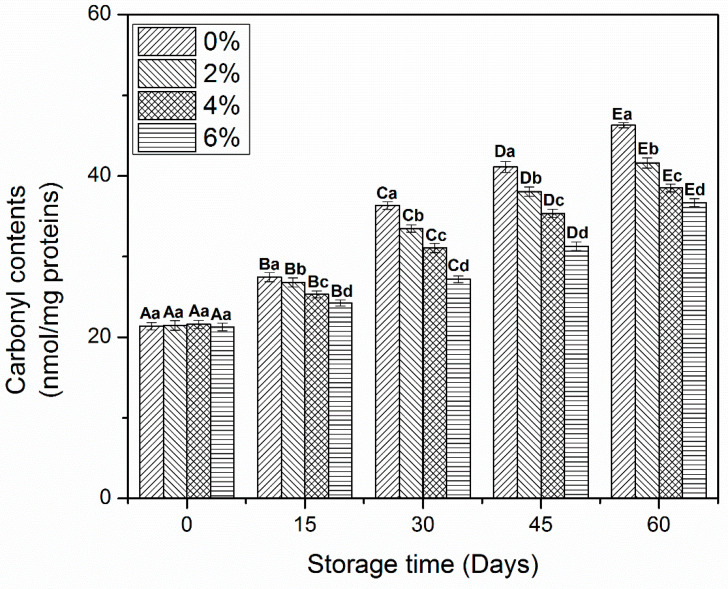
Carbonyl contents of myofibrillar protein treated with different concentrations of OVA-KC during 60 days of frozen storage at −18 °C. Capital letters (A–E) refers to the significant differences in similar treatments within the different storage times (*p* < 0.05). Lowercase letters (a–d) refers to the significant difference in the same frozen storage period of different concentrations (*p* < 0.05).

The determination of carbonyls is important to evaluate the oxidative alterations in muscle protein during frozen storage [10]. The carbonyl contents of MP treated with OVA-KC are shown in Figure 1. All samples treated with OVA-KC reported a significant increase (*p* < 0.05) in carbonyls during the frozen storage. During the first 15 days of storage, no significant differences were found between MP samples. Then, the values of carbonyls started to increase until the end of storage, observing the maximum increase in 0% OVA-KC from day 15 to day 60. MP treated with 2% OVA-KC showed an increase in carbonyls but a better stability was found than in control MP. On the other hand, samples, treated with higher concentrations of OVA-KC (4% and 6%), also showed an increase in protein oxidation, protein carbonylation was significantly lower than in control samples. Within dose effect, OVA-KC added at 6% showed the highest inhibitory effect on and protein oxidation after day 60 of frozen storage.

The rise in carbonyls in MP during frozen storage could be increased due to amounts of water-soluble proteins, denaturation and protein aggregation. Moreover, this increase of the oxidation degree could also reduce the functional, structural, and gelling characteristics of protein-based products [25]. Walayat, et al. [19] reported that the addition of EWP/CD reduced the rate of oxidation in muscle proteins by binding at the functional sites of proteins though proper cross-linking and prevented the freeze-induced changes in amino acid side chains that are most susceptible to oxidative changes, inhibiting their conversion in carbonyls. Shui et al. [26] reported that the addition of carrageenan significantly reduced the increase in carbonyl contents of shrimp protein due to its effectiveness against formation of free radicals and hydro-peroxides than the sodium pyrophosphate added proteins during frozen storage. Furthermore, the incorporation of EWP-XO in MP also prevented the increased carbonyls by inhibiting oxidation in proteins [4]. Furthermore, the current study proposes the addition of OVA-KC as an effective approach to prevent the formation of carbonyls due to their proper cross-linking with amino acid molecules, which can reduce protein–water and protein–protein interactions during frozen storage.

### 3.3. Ca_2+_-ATPase Activity

**Figure 2:**
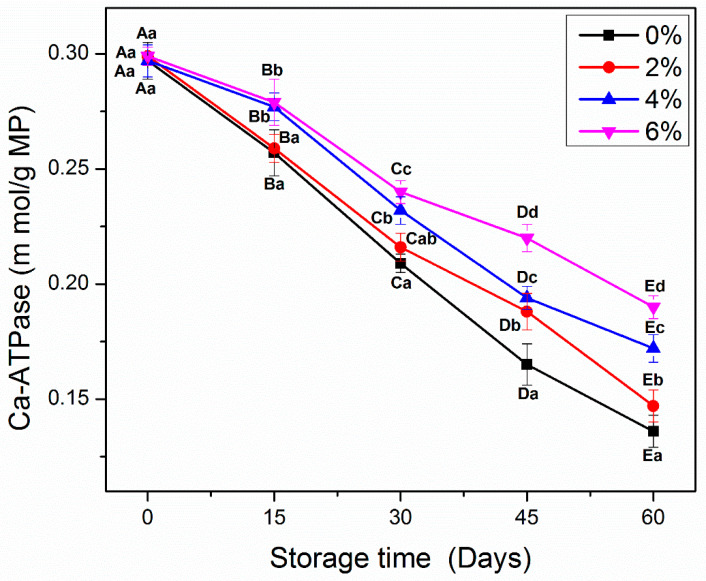
Ca^2+^-ATPase activity of myofibrillar protein treated with different concentrations of OVA-KC during 60 days of frozen storage at −18 °C. Capital letters (A–E) refer to the significant differences in similar treatments within the different storage times (*p* < 0.05). Lowercase letters (a–d) refers to the significant difference in the same frozen storage period of different concentrations (*p* < 0.05).

Ca^2+^-ATPase activity is another key analysis to examine the integrity of protein molecules during frozen storage [2,27,28]. Figure 2 shows the Ca^2+^-ATPase activity of all MP samples during frozen storage. From day 0 to 60, all MP samples showed a significant decline (*p* < 0.05) in the Ca^2+^-ATPase activity. At the beginning of storage, no significant differences were observed between control and MP treated with different concentrations of OVA-KC. As expected, control samples showed the greatest decrease in Ca_2+_-ATPase activity at the end of frozen storage. MP treated with 2% OVA-KC also displayed a significant decline (*p* < 0.05) in Ca^2+^-ATPase, but less marked than that observed in the control MP at the end of frozen storage. In contrast, a lesser decrease was observed in samples treated with 4% OVA-KC and 6% OVA-KC, which showed better stability against control and 2% OVA-KC samples. The differences between the highest doses of OVA-KC were more visible after day 45 onwards (Figure 2), being 6% OVA-KC the one that showed the best stability of Ca^2+^-ATPase activity.

This loss of Ca^2+^-ATPase activity could be due to the irregular formation and growth of ice crystals that led to the denaturation of myosin molecules [3]. Moreover, the change in Ca^2+^-ATPase activity is also critically interrelated with tertiary structural changes and sulfhydryl contents, which can be catalyzed by a weak intermolecular binding interaction and the oxidation in myosin rod region [13]. Moreover, KC showed better cryoprotective properties against the oxidative changes than the conventional cryoprotectants including sucrose, sorbitol, and glucose, which are previously reported by Zhang et al. [2]. Zhang et al. [2] reported that the addition of KC and alginate significantly improved the Ca^2+^ATPase activity of MP during frozen storage than the sucrose (positive control), which could be due to the hydrophilic hydroxyl groups at functional sites of KC and alginate. 

The addition of a cryoprotective mixture of EWP/βCD could reduce the drop in Ca^2+^-ATPase by stabilizing the surface tension of water, improving cross-linking between protein molecules and inhibiting protein–water interactions [19]. The results of the present study support this fact, since OVA-KC could be used efficiently in the prevention of Ca^2+^-ATPase decrease.

### 3.4. Sulfhydryl Content

MP is composed of sulfhydryl (SH) groups, which are more prone to oxidation and converted to disulfide bonds during freeze-induced changes. These changes mainly occur in the myosin rod region and have a close relationship with the reduction of Ca^2+^-ATPase activity. In addition, SH content is reduced by prolonged oxidative changes during frozen storage [10,29].

**Figure 3:**
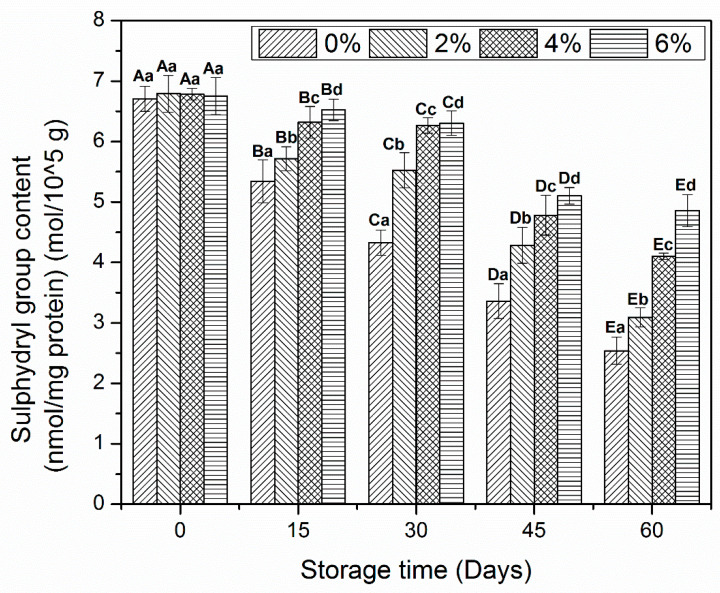
Sulfhydryl contents myofibrillar protein treated with different concentrations of OVA-KC during 60 days of frozen storage at −18 °C. Capital letters (A–E) refers to the significant differences in similar treatments within the different storage times (*p* < 0.05). Lowercase letters (a–d) refers to the significant difference in the same frozen storage period of different concentrations (*p* < 0.05).

The reduction in SH contents of MP samples treated with OVA-KC is shown in Figure 3. No prominent changes were observed between MP samples on day 0. The decline of SH content started after day 15 of storage. Control MP sample and 2% OVA-KC were those that showed a notable decrease in SH at the end of frozen storage. This decrease was reduced as the added concentration of OVA-KC increased. Therefore, a better stability was observed in samples treated with 4% OAV-KC and 6% OAV-KC. The difference between these samples was more marked from day 45 onwards. However, 6% OAV-KC was the one that best that inhibited the loss of SH contents after day 60 of frozen storage.

The formation of disulfide contents during oxidative changes could be the reason for the decrease in SH groups [30,31]. Furthermore, these changes are related to the alteration in the myosin head region, which is very sensitive to oxidative modifications that lead to the loss of structural and functional properties [32]. As discussed earlier, SH groups and Ca^2+^-ATPase are directly associated with change in myosin [33]. Therefore, the decline in SH groups coincides with the decrease in Ca^2+^-ATPase activity (shown in Figure 2). Moreover, the addition of cryoprotectants protects the myosin head region by inhibiting the oxidative changes and prompting the proper cross-linking among amino acid molecules. In addition, incorporation of carrageenan oligosaccharides in shrimp protein effectively enhanced the stability of SH groups after 120 days of frozen storage. Besides that, significant decline was found in the protein added with sodium pyrophosphate [26]. KC could reduce the decline in SH groups owing to its antioxidative and radical scavenging abilities toward DPPH, OH and radicals [10]. Therefore, it can be concluded from the current results that the addition of OVA-KC prevented the formation of disulfide bridges by making strong interaction at the functional sites of amino acid side chains.

### 3.5. Dityrosine Content

**Figure 4:**
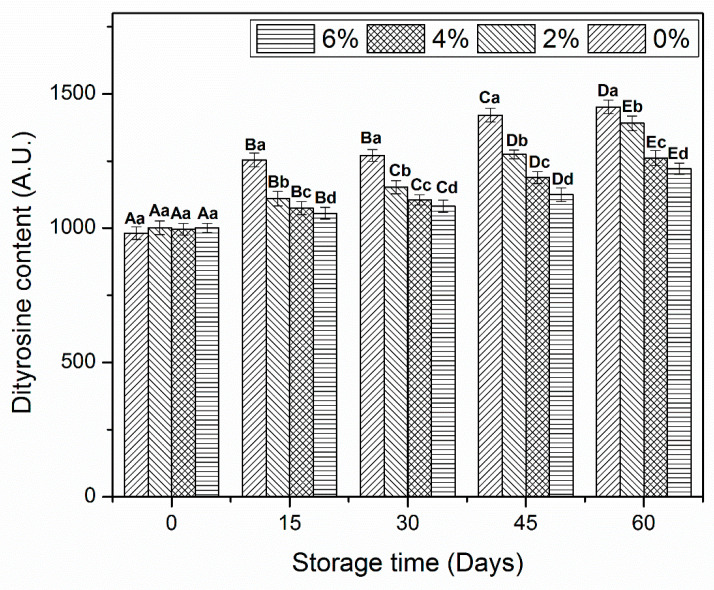
Dityrosine contents of myofibrillar protein treated with different concentrations of OVA-KC during 60 days of frozen storage at −18 °C. myofibrillar protein treated with different concentrations of OVA-KC during 60 days of frozen storage at −18 °C. Capital letters (A–E) refer to the significant differences in similar treatments within the different storage times (*p* < 0.05). Lowercase letters (a–d) refers to the significant difference in the same frozen storage period of different concentrations (*p* < 0.05).

Dityrosine allows to determine the oxidative changes in amino acids caused by alteration in L-tyrosine molecules [34]. All MP samples revealed a significant increase in dityrosine content after day 60 of frozen storage (Figure 4). As it occurred in the previous analysis, no significant difference was found at the beginning of storage. The control MP revealed a remarkable increase in dityrosine content after day 15 of storage. Although, samples treated with OVA-KC also showed a significant increase, it was less pronounced. At the end of storage, 6%OVA-KC displayed the smallest increase in dityrosine contents.

The increase in dityrosine content could be due to the weak bonding interactions between amino acid molecules. In addition, it could be also associated with MP oxidation, resulting in reduced SH groups (Figure 3.) and Ca^2+^-ATPase (Figure 2.) activity [35]. In addition, these results are totally in agreement with Zhang et al. [36] who reported that addition of KC (3%) significantly restricted the increase in dityrosine content than the KC (1%) and control shrimp protein samples during frozen storage. Meanwhile, these results, which indicated the formation of new cross-linking in shrimp proteins after the incorporation of oligosaccharides are consistent with the Walayat et al. [9]. Moreover, Colombo, et al. [37] stated that the MP oxidative changes increased in similar ways, such as increased dityrosine, formation of disulfide bindings and protein denaturation. Our results indicated that OVA-KC efficiently restricted the increase in dityrosine content by inhibiting oxidative changes and forming proper cross-linking between the amino acids during frozen storage.

### 3.6. Surface Hydrophobicity (S_0_)

Alteration in surface hydrophobicity (S_0_) characterizes the conformational modifications in the protein structure [38]. The changes in the surface hydrophobicity (S_0_) of MP treated with different concentrations of OVA-KC are shown in Table 1. All samples showed a notable increased of S_0_, which indicates the exposure and conformational changes in the protein molecules. Therefore, it seems that conformational changes of MP are directly affected by frozen storage.

During the frozen storage, control MP (0%) showed a prominent increase in S_0_ compared to those found in samples treated with OVA-KC. The increase in surface hydrophobicity could be due to the formation of hydrophobic interaction between the exposed hydrophobic molecules, which results in increased protein denaturation and aggregation [31]. Walayat et al. [19] stated that the increase in S_0_ is ascribed to the exposure and unfolding of aromatic and hydrophobic amino acids during frozen storage. 

From the current results, OVA-KC restricted the increase in S_0_. Samples treated with the highest concentration of OVA-KC (6%) showed a delayed increase in surface hydrophobicity than samples added with 2% and 4% of OVA-KC (72.89 vs. 74.29 and 84.65, respectively). This inhibited increase in S_0_ could be due to the suppressed oxidation and denaturation of proteins with the addition of OVA-KC. This is probably due to the better hydrophilic-hydrophilic interaction between OVA-KC and protein molecules. In addition, the increase in S^0^ could be due to the exposure of the hydrophobic interior resulting in protein denaturation and aggregation [39]. Therefore, the addition of OVA-KC restricted the exposure of hydrophobic residue and resulting in the formation of hydrophobic interaction clusters and aggregates. In the meantime, crystallization could also play a key role in protein denaturation by disrupting the hydrate layer around the polar residues. Zhang et al. [2] reported that addition of KC (3%) increased the S_0_ of the shrimp proteins during frozen storage of 120 days than the samples added with KC (1% and control) by restricting the protein denaturation and aggregation. It is also possible that the addition of KC in protein can increase the hydrophilic–hydrophilic interactions. In addition, traditional cryoprotectants—such as sucrose, sorbitol, βCD, and xylooligosaccharides—could be a protective shield against protein oxidation during frozen storage [19]. Some previous studies also reported that addition of sucrose and sorbitol can also efficiently inhibited the increase in S_0_ but the oligosaccharides, such as KC and alginates, are highly recommended owing to their low molecular weight, ionic and hydrogen bindings [23]. This could be because the cryoprotectants binds to one of the functional sites of protein molecules through ionic and hydrogen bonds and provides an antioxidant shield against oxidation and denaturation of proteins [18].

### 3.7. Fluorescence Intensity (FI)

Fluorescence intensity (FI) is a well-known technique used for the determination of the tertiary structural changes in proteins during frozen storage. The FI is sensitive to the presence of tryptophan (Trp) residues and polarity of the micro-environment [3]. Therefore, the existence of hydrophobic and Trp residues in proteins reveals higher FI and the change in FI exhibits alteration in tertiary structural properties [40]. 

The FI and wavelength of all MP samples with or without OVA-KC are shown in Table 2 and Table 3. All MP samples showed a significant decline (*p* < 0.05) in FI at the end of frozen storage. In control samples, FI decreased remarkably from 272.23 to 185.57 A.U., while samples treated with the lowest concentration of OVA-KC (2%) exhibited a minor decline from 273.98 A.U. to 192. 5 A.U as shown in Table 2. This reduction indicated the exposure of Trp residues to the aqueous environment and denaturation of protein molecules [41]. In contrast, samples treated with the highest concentrations of OVA-KC (4% and 6%) exhibited a greater delay in this decline (206.82 and 211.59 A.U., respectively). During the analyses, 6% OVA-KC showed greater stability, in this case against change in Trp residues, and allowed the exposure of indole side chains to the polarity of the microenvironment. Meanwhile, as depicted from Table 3 fluorescence emission of grass carp MP was found between 330 and 340 nm. Therefore, the addition of cryoprotectants would reduce the conformational changes in amino acids by providing proper cross-linking and preventing these from protein denaturation [19]. From our results, we can conclude that the increased concentration of OVA-KC improved the tertiary structural properties by restricting the oxidation due to the formation of water-soluble molecules, protein–protein and protein–water interactions. These results are further verified by surface hydrophobicity (Table 1), but also according to the stable SH groups (Figure 3) and Ca^2+^-ATPase activity (Figure 2).

### 3.8. Circular Dichroism

**Figure 5:**
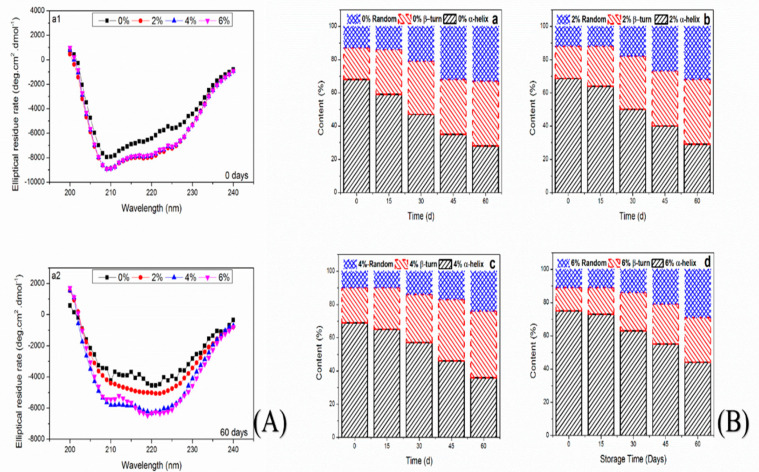
Far-UV CD spectra of myofibrillar proteins treated with different concentrations of OVA-KC during 60 days of frozen storage at −18 °C. (**A**) represent change in CD spectra from 200 to 240 nm (**B**) represent change in secondary structural properties.

The change in secondary structural properties of MP was determined by circular dichroism (CD), which can easily determine the modifications in α-helices, β-turns, and random coils [42]. The secondary structural alterations of samples treated with OVA-KC are shown in Figure 5A,B. CD spectra showed two bands at 208 and 220 nm ascribed to the change in α-helix contents [43]. The alteration in these bands would be valuable to examine the change in α-helix properties due to its presence in the myosin region, since α-helix comprised of a total 95% of myosin [44]. 

During analysis, all samples showed declines at 208 and 220 nm. At day 0 of analysis, all samples showed a similar trend, which became prominent after day 60 of frozen storage. Control samples exhibited a considerable decline in both regions, which corresponded to the prominent change in α-helix from 68% to 28% and β-turns increased from 19% to 39%, which is usually stabilized by the hydrogen interactions between the peptide [45]. With the extended frozen storage temperature, β-turns significantly increased, which is indicates disrupted hydrogen interactions between the peptides and amino acid chains resulting in unfolded and aggregated proteins [46]. Meanwhile, Walayat et al. [19] also reported that the increase in the β structure and random coils could be due to the proteins that undergo aggregation, rearrangement and unfolding. Moreover, Li et al. [45] reported that the frozen storage reduced the structural properties of MP resulting due to the formation of protein aggregates. MP treated with different concentrations of OVA-KC also displayed a decline in α-helix contents (Figure 5), which become sharper with increased concentrations of OVA-KC. MP treated with a higher concentration of OVA-KC (6%) displayed mild decline at both bands (208 and 220 nm). At the end of the frozen storage, α-helix decreased from 75% to 44%, while β-turns increased from 14% to 27% (Figure 5B), which is ascribed to the secondary structural changes. Usually, the decrease in α-helix is linked with the change in amide –NH and hydrogen bindings, which is a well-known force that keeps α-helix content stable [47]. The increase in beta structural changes could be due to the exposure of hydrophobic residues with the water molecules leading. Meanwhile, the crystallization can also be chief factor, which cause from the free water molecules resulting into the denaturation and aggregation of myosin [44]. Moreover, secondary structural properties are also reduced due to weak hydrogen bindings between the amino acid side chains, which are closely associated with alteration in myosin, and a key component in improved gelling and functional properties of protein products [48]. In addition, disulfide bonds formed during the oxidation process of SH groups, demonstrating that increasing disulfide groups could reduce myosin conformational changes during freeze storage. Therefore, our current results are related to the reduction of SH groups (Figure 3), which was controlled with the addition of OVA-KC. In this regard, Walayat et al. [4] reported that the addition of EWP/XO significantly reduced the secondary structural changes by inhibiting the oxidative changes in myosin head. Liu et al. [14] reported that the addition of konjac oligosaccharide improved the secondary structural properties surimi gel. Walayat, et al. [4] analyzed that the addition of EWP and βCD mixture in MP prevented the decline secondary structural properties resulting due to the oxidative changes during the frozen storage of 60 days.

### 3.9. Intermolecular Interaction Force

Intermolecular forces play a significant role in the mechanical, structural and gelling properties of MP. These are based on the type of protein–protein and protein–water interactions. Hydrophobic bindings are important for gelling characteristics [49]. While, ionic and hydrogen bindings influence the WHC and textural attributes of protein-based products [40]. 

**Figure 6:**
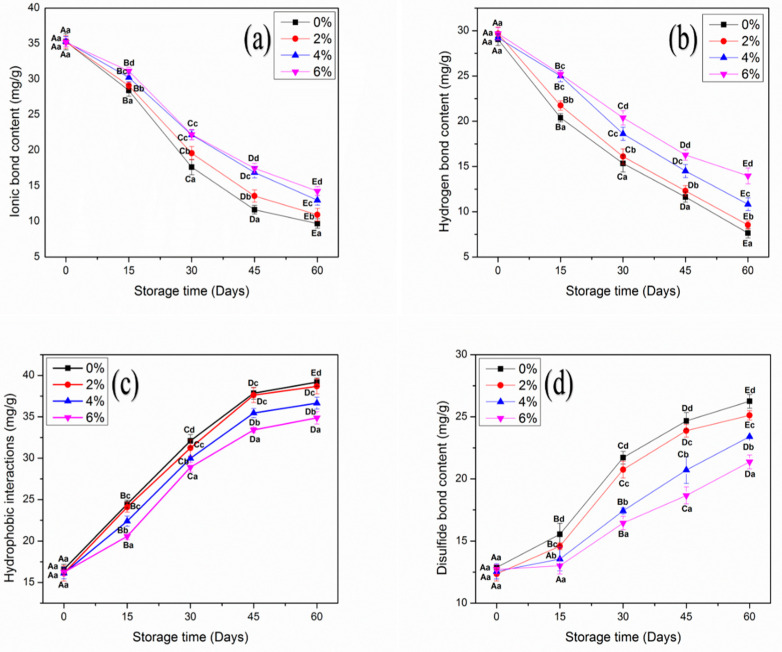
The content of ionic bond (**a**), hydrogen bond (**b**), hydrophobic interaction (**c**), and disulfide bond (**d**) of myofibrillar protein treated with different concentrations of OVA-KC during 60 days of frozen storage at −18 °C. Capital letters (**A**–**E**) refer to the significant differences in similar treatments within the different storage times (*p* < 0.05). Lowercase letters (**a**–**d**) refers to the significant difference in the same frozen storage period of different concentrations (*p* < 0.05).

The changes in intermolecular interaction forces of MP samples treated with OVA-KC are shown in Figure 6. All MP samples reported significant (*p* < 0.05) alterations. At day 0 analysis, no significant differences were found between samples for ionic and hydrogen interactions. The change in binding interactions was initiated on day 15 in all MP samples. The control sample showed a major decline in ionic and hydrogen binding interactions after day 60 of frozen storage. The decline was reduced with the addition of OVA-KC. In this regard, 2% OVA-KC showed a similar trend but better stability than control. In the case of 4% OVA-KC and 6% OVA-KC, no significant changes were observed from day 30 to 45. At the end of storage, 6% OVA-KC showed the best results, with a more restricted decrease.

The ionic bindings in MP were primarily formed by the steric hindrance of the protein–protein and protein–water interactions produced by the net negative surface charge. Ice crystal formation decreases the activity of the protein and reduces the surface charge, subsequently decreasing ionic binding interactions, which are attributed to protein aggregation and denaturation. Moreover, the decline in hydrogen binding could be due to an increase in oxidative changes, which also corresponds to the reduction of gelling and WHC properties [49]. During the gelation process, improved SH groups can influence the intermolecular binding interactions and assist in the formation of a more compact and well-established three-dimensional gel network [50,51]. 

On the other hand, hydrophobic interactions and disulfide bridges increased with prolonged frozen storage (Figure 6). Control MP showed a significant increase in both bindings, while samples with 6% OVA-KC significantly restricted the increase in hydrophobic and disulfide formations. These findings are completely consistent with previous findings of improved surface hydrophobicity (Table 1), dityrosine fluorescence intensity (Table 2), and SH groups (Figure 3). In addition, hydrophobic interactions and disulfide bridges of MP could influence the disordered arrangement and weak cross-linking of amino acid side chains, resulting in reduced structural and functional properties [52]. Thus, it can be recommended from the results obtained that the addition of OVA-KC efficiently enhanced the intermolecular binding interactions by reducing oxidative changes in protein molecules.

## 4. Conclusions

This study suggested that the incorporation of OVA-KC improved the functional and structural properties of MP by inhibiting the oxidative changes. Prolonged frozen storage significantly reduced the SH contents and Ca^2+^-ATPase activity. In addition, increased carbonyl and surface hydrophobicity were analyzed, related to irregular unfolding, weak cross-linking and oxidative changes in protein molecules. Besides that, the addition of OVA-KC efficiently reduced these changes by inhibiting the protein oxidation. Moreover, 6% OVA-KC improved the secondary and tertiary structural properties by providing a protective shield to myosin globular head. Incorporation of OVA-KC also improved intermolecular binding interactions (ionic, hydrogen, hydrophobic, and disulfide) linked with functional, gelling, and structural properties. Overall, MP treated with 6% of OVA-KC showed better stability against oxidative and structural changes during 60 days of frozen storage. Therefore, it can be concluded from the current results that OVA-KC could be a better alternative to traditional cryoprotectants in seafood-based products and in the surimi industry.

## Figures and Tables

**Table 1:** Protein solubility and surface hydrophobicity of myofibrillar proteins treated with different concentrations of OVA-KC during 60 days of frozen storage at −18 °C.

Protein Solubility (%)				Surface Hydrophobicity (S_0_)			
Days 0%	2%	4%	6%	0%	2%	4%	6%
0 53.90 ± 0.64^Aa^	53.96 ± 0.59^Aa^	53.78 ± 0.72^Aa^	53.82 ± 0.67^Aa^	43.26 ± 0.51^Aa^	43.54 ± 0.62^Aa^	43.47 ± 0.51^Aa^	43.61 ± 0.76^Aa^
15 39.97 ± 0.52^Ba^	37.35 ± 0.54^Bb^	41.26 ± 0.48^Bc^	42.66 ± 0.52^Bd^	67.45 ± 0.45^Ba^	64.81 ± 0.55^Bb^	59.83 ± 0.66^Bc^	57.22 ± 0.64^Bd^
30 27.32 ± 0.61^Ca^	29.11 ± 0.61^Cb^	32.61 ± 0.61^Cc^	34.24 ±0.81^Cd^	73.41 ± 0.55^Ca^	71.62 ± 0.54^Cb^	67.52 ± 0.63^Cc^	63.74 ± 0.60^Cd^
45 19.13 ± 0.47^Da^	21.77 ± 0.83^Db^	26.44 ± 0.64^Dc^	28.45 ± 0.72^Dd^	77.21 ± 0.54^Da^	76.35 ± 0.87^Db^	70.08 ± 0.58^Dc^	68.33 ± 0.77^Dd^
60 11.27 ± 0.55^Ea^	12.42 ± 0.73^Eb^	16.23 ± 0.70^Ec^	18.12 ± 0.65^Ed^	85.32 ± 0.63^Ea^	84.65 ± 0.66^Eb^	74.29 ± 0.70^Ec^	72.89 ± 0.64^Ed^

Upper case letters (A–E) represent the significant difference (*p* < 0.05) in same concentration in column. Lower case letters (a–d) represent the significance difference (*p* < 0.05) of different concentrations in row. All samples and values were taken and analyzed in triplicates.

**Table 2:** Intrinsic fluorescence intensity of myofibrillar proteins treated with different concentrations of OVA-KC during 60 days of frozen storage at −18 °C.

	Intrinsic Fluorescence Intensity			
Days	0%	2%	4%	6%
0	272.23 ± 0.58^Aa^	273.98 ± 1.07^Aa^	274.13 ± 1.44^Aa^	280.6 ± 1.04^Ab^
15	220.77 ± 0.31^Ba^	227.08 ± 4.24^Bb^	239.83 ± 4.52^Bc^	249.84 ± 3.88^Bd^
30	205.4 ± 4.95^Ca^	216.1 ± 9.74^Cb^	232.03 ± 8.78^Cc^	240.6 ± 5.49^Cd^
45	188.83 ± 0.85^Da^	206.33 ± 4.67^Db^	228.03 ± 6.43^Dc^	239.17 ± 0.49^Dd^
60	185.57 ± 2.53^Da^	192.5 ± 4.07^Eb^	206.82 ± 6.44^Ec^	211.59 ± 9.28^Ed^

Upper case letters (A–E) represent the significant difference (*p* < 0.05) in same concentration in column. Lower case letters (a–d) represent the significance difference (*p* < 0.05) of different concentrations in row. All samples and values were taken and analyzed in triplicates.

**Table 3:** Fluorescence wavelength of myofibrillar proteins treated with different concentrations of OVA-KC during 60 days of frozen storage at −18 °C.

	Fluorescence Wavelength			
Days	0%	2%	4%	6%
0	334.48 ± 0.40^Aa^	334.01 ± 0.53^Aa^	333.82 ± 0.30^Aab^	334.03 ± 0.20^Ab^
15	335.51 ± 0.36^Aa^	335.67 ± 0.44^Ba^	336.87 ± 0.18^Bb^	336.34 ± 0.81^Bb^
30	333.18 ± 1.24^Ba^	334.23 ± 0.21^Ba^	334.27 ± 0.32^Ca^	334.82 ± 1.22^Cb^
45	332.53 ± 0.55^BCa^	332.81 ± 0.39^Ca^	333.79 ± 0.51^Cab^	333.05 ± 0.74^Dc^
60	331.91 ± 0.67^Ca^	331.48 ± 1.53^Da^	333.15 ± 0.36^Cb^	333.26 ± 0.31^Eb^

Upper case letters (A–E) represent the significant difference (*p* < 0.05) in same concentration in column. Lower case letters (a–d) represent the significance difference (*p* < 0.05) of different concentrations in row. All samples and values were taken and analyzed in triplicates.

## Data Availability

Data is contained within the article.
